# Protection against the Metabolic Syndrome by Guar Gum-Derived Short-Chain Fatty Acids Depends on Peroxisome Proliferator-Activated Receptor γ and Glucagon-Like Peptide-1

**DOI:** 10.1371/journal.pone.0136364

**Published:** 2015-08-20

**Authors:** Gijs den Besten, Albert Gerding, Theo H. van Dijk, Jolita Ciapaite, Aycha Bleeker, Karen van Eunen, Rick Havinga, Albert K. Groen, Dirk-Jan Reijngoud, Barbara M. Bakker

**Affiliations:** 1 Center for Liver, Digestive and Metabolic Diseases, Department of Pediatrics & Systems Biology Center for Energy Metabolism and Ageing, University of Groningen, University Medical Center Groningen, Groningen, The Netherlands; 2 Department of Laboratory Medicine, University of Groningen, University Medical Center Groningen, Groningen, The Netherlands; 3 Netherlands Consortium for Systems Biology, Amsterdam, The Netherlands; 4 Top Institute Food and Nutrition, P.O. Box 557, 6700 AN Wageningen, The Netherlands; East Tennessee State University, UNITED STATES

## Abstract

The dietary fiber guar gum has beneficial effects on obesity, hyperglycemia and hypercholesterolemia in both humans and rodents. The major products of colonic fermentation of dietary fiber, the short-chain fatty acids (SCFAs), have been suggested to play an important role. Recently, we showed that SCFAs protect against the metabolic syndrome via a signaling cascade that involves peroxisome proliferator-activated receptor (PPAR) γ repression and AMP-activated protein kinase (AMPK) activation. In this study we investigated the molecular mechanism via which the dietary fiber guar gum protects against the metabolic syndrome. C57Bl/6J mice were fed a high-fat diet supplemented with 0% or 10% of the fiber guar gum for 12 weeks and effects on lipid and glucose metabolism were studied. We demonstrate that, like SCFAs, also guar gum protects against high-fat diet-induced metabolic abnormalities by PPARγ repression, subsequently increasing mitochondrial uncoupling protein 2 expression and AMP/ATP ratio, leading to the activation of AMPK and culminating in enhanced oxidative metabolism in both liver and adipose tissue. Moreover, guar gum markedly increased peripheral glucose clearance, possibly mediated by the SCFA-induced colonic hormone glucagon-like peptide-1. Overall, this study provides novel molecular insights into the beneficial effects of guar gum on the metabolic syndrome and strengthens the potential role of guar gum as a dietary-fiber intervention.

## Introduction

The growing prevalence of diseases clustered in the metabolic syndrome is accompanied by a shift in diet in Western and developing countries from a traditional low-calorie diet with high-fiber and low-fat content towards a high-calorie diet with low-fiber and high-fat content [1,2]. Epidemiological studies revealed an inverse correlation between dietary fiber intake and the metabolic syndrome [3], suggesting that fiber supplementation to the diet may be beneficial. Indeed, a variety of studies have shown that dietary fiber intervention decreased obesity and insulin resistance in both healthy and metabolic syndrome patients (reviewed by Galisteo *et al*. [1]). In randomized controlled clinical trials a 6-week supplementation of one of the most promising fibers (guar gum) reduced fasting plasma glucose and insulin levels, and increased insulin sensitivity in healthy and type 2 diabetes humans [4,5]. Prolonging the guar gum supplementation to 8 weeks also led to a decrease in fat mass and body weight in overweight subjects [6,7]. The beneficial effects of dietary fibers work through the intestinal microbiota [8]. The main products of intestinal bacterial fermentation of dietary fiber are the short-chain fatty acids (SCFAs), of which acetate, propionate and butyrate are most abundant [9]. SCFAs have been suggested to play a major role in the dietary fiber-induced beneficial effects. We showed that dose-dependent effects of guar gum-supplementation on body weight and insulin sensitivity correlate with the rate of SCFA uptake by the host, but not with their cecal concentration [10], suggesting that fiber-derived SCFAs need to be taken up to exert their full physiological effect.

When any of the three individual SCFAs is supplied directly via the diet, they are highly efficacious in protection against the high-fat diet-induced metabolic syndrome (*i*.*e*. insulin resistance and obesity) [11–13]. In these studies, SCFAs had no effect on glucose tolerance. We recently elucidated the underlying mechanism: dietary SCFAs repress peroxisome proliferator-activated receptor (PPAR) γ expression, subsequently increasing mitochondrial uncoupling protein (UCP) 2 expression and AMP/ATP ratio, leading to the activation of AMP-activated protein kinase (AMPK) and culminating in enhanced oxidative metabolism in both liver and adipose tissue [13].

In this study we investigate whether guar gum exerts its effects on body weight, insulin sensitivity and glucose tolerance via the same signaling cascade as SCFAs, as should be expected when the produced SCFAs are the main molecular mediators of the guar gum-induced effects. In addition, we reveal an additional role for the guar gum-derived SCFA-induced colonic hormone glucagon-like peptide-1.

## Materials and Methods

### Ethics Statement

The national and institutional guidelines for the care and use of animals were followed, and the experimental procedures were reviewed and approved by the Ethics Committees for Animal Experiments of the University of Groningen, The Netherlands (ethics registration code 5887). All efforts were made to minimize suffering.

### Animals and Experimental Design

Male C57Bl/6J mice (Charles River, L’Arbresle Cedex, France), 2 months of age, were housed in a light- and temperature-controlled facility (lights on 6:30 a.m. to 6:30 p.m., 21°C) and had free access to water and food. They were fed a high-fat semi-synthetic diet (D12451, Research Diet Services, Wijk Bij Duurstede, The Netherlands), which (unlike very similar diets from other suppliers) contained 10% corn starch. In the experimental groups 10% (w/w) guar gum (Viscogum^TM^ MP 41230, Cargill, United States) replaced the equivalent amount of corn starch. All analyses were done in eight mice per group, unless indicated otherwise.

### Plasma and tissue sampling

Mice were fasted from 6–10 a.m. Blood glucose concentrations were measured using a EuroFlash meter (Lifescan Benelux, Belgium). Mice were subsequently sacrificed by cardiac puncture under isoflurane anesthesia. DPP IV inhibitor (DPP4, Millipore) was added, blood was centrifuged (4000 x *g* for 10 min at 4°C) and plasma was stored at -80°C. Liver was quickly removed, snap-frozen in liquid nitrogen and stored at -80°C. Epididymal fat pads were weighed. Cecal content and cecum were quickly removed, snap-frozen in liquid nitrogen and stored at -80°C. Plasma NEFA concentrations were determined using a commercially available kit (Roche Diagnostics, Germany). Plasma insulin (ALPCO Diagnostics, United States), PYY (ALPCO Diagnostics, United States) and GLP-1 levels (Merck Millipore, United States) were determined using ELISA and HOMA-IR was calculated (IR = fasting insulin mU/L x fasting glucose mM ÷ 22.5). Hepatic TG content was determined using a commercially available kit (Roche) after lipid extraction [14].

Lipogenesis was determined from the incorporation of [1-^13^C]-acetate into palmitate by providing 2% (w/v) [1-^13^C]-acetate in drinking water for 24h as described previously [15]. Fatty acid ß-oxidation capacity was measured in fresh liver and adipose homogenates according to Hirschey et al. [16]. Briefly, tissue was homogenized in sucrose/Tris/EDTA buffer, incubated for 30 min in the reaction mixture (pH 8.0) containing [1-^14^C]palmitic acid, and trapped [^14^C]CO_2_ was measured. Adenine nucleotide concentrations were determined by HPLC according to Miller et al. [17].

### Indirect calorimetry

Oxygen consumption, energy expenditure, respiratory exchange ratio (RER), food intake, and activity patterns were measured simultaneously for six mice per group using the Comprehensive Laboratory Animal Monitoring System and Software (TSE Systems GmbH, Germany). The energy balance was determined by measuring the energy content [18] of diet and dried, homogenized feces using a bomb calorimeter (CBB 330, standard benzoic acid 6320 cal g^-1^, BCS-CRM no.90N).

### Glucose and insulin tolerance

Whole body glucose tolerance was determined by intraperitoneal injection of 2 g glucose per kg body weight after an overnight fast of 9h. Whole body insulin tolerance was measured by intraperitoneal injection of insulin (NovoRapid) at 0.75 units/kg body weight after a 4h fast. Hyperinsulinemic-euglycemic clamp studies were performed as previously described [19].

### Oxygen consumption rates in liver mitochondria

Mitochondria were isolated from fresh liver tissue according to Mildaziene *et al*. [20]. The rates of oxygen consumption in isolated liver mitochondria were measured at 37°C using a two-channel high-resolution Oroboros oxygraph-2k (Oroboros, Innsbruck, Austria) with palmitoyl-CoA and malate as substrates in mitochondrial respiration medium [21]. Maximal ADP-stimulated oxygen consumption (state 3) was achieved by adding 1.5 U ml^-1^ hexokinase, 12.5 mM glucose and 1 mM ATP. The resting state oxygen consumption rate (state 4) was determined after blocking ADP phosphorylation with 1.25 μM carboxyatractyloside. The respiratory control ratio (RCR) was calculated by dividing the oxygen consumption rate in state 3 by the oxygen consumption rate in state 4.

### Determination of SCFA concentrations

Cecal concentrations of SCFAs were measured as previously described [22]. In short, cecum content was centrifuged and 25 μl of supernatant was spiked with 25 μl of internal standard (17.3 mM hydroxyisocapronic acid) and 5 μl of 20% 5-sulfosalicyclic acid. After a 10 min centrifugation the supernatant was acidified with 2.5 μl 37% HCl and SCFA were extracted with 2 ml diethylether. Derivatization was performed overnight with 500 μl supernatant and 50 μl of N-tert-Butyldimethylsilyl-N-methyltrifluoroacetamide (MTBSTFA). SCFAs were measured in an Agilent 5975 series GC/MSD (Agilent Technologies). The gas chromatograph was equipped with a ZB-1 column (Phenomenex). Mass spectrometry analysis was performed by electron capture negative ionization with methane as the moderating gas.

### Cecal SCFA infusion experiments

Mice were fed the HFD without guar gum for 6 weeks. Cecal SCFA infusions were performed as described previously [22]. Briefly, mice were equipped with a permanent cecum catheter and allowed a recovery period of at least 5 days. On the day of the experiment, the mice were individually housed and fasted from 6:00 to 10:00 a.m. All infusion experiments were performed in conscious, unrestrained mice. Four different groups of each 8 mice received a 140 mM phosphate-buffered saline (140 mM NaCl, 10 mM sodium phosphate at pH 5.8), a 140 mM sodium acetate (S2889; Sigma), a 140 mM sodium propionate (P1880; Sigma) or a 140 mM sodium butyrate (303410; Sigma) solution infused via the cecum catheter at a rate of 0.2 ml/h for 6h. The infusion rate of SCFA was based on the recommended intake of dietary fiber for humans of 38 g/day/human, which results in approximately 380 mmol SCFAs/day/human [23,24]. When calculated for mice, this corresponds to 170 μmol SCFAs/day/mouse. By infusing 140 mM SCFA directly into the cecum at a rate of 0.2 ml/h for 6h, a total amount of 168 μmol SCFA was given per mouse. After 6h of infusion, animals were terminated by cardiac puncture under isoflurane anesthesia. Cecum was removed quickly, freeze-clamped, and stored at -80°C. Blood was centrifuged (4000 x *g* for 10 min at 4°C) and plasma was stored at -80°C.

### Gene expression levels and immunoblot analysis

RNA was extracted from mouse livers using Tri reagent (Sigma-Aldrich, St. Louis, MO) and converted into cDNA by a reverse transcription procedure using M-MLV and random primers according to the manufacturer’s protocol (Sigma-Aldrich). For quantitative PCR (qPCR), cDNA was amplified using the appropriate primers and probes. Taqman RT-PCR primer and probe were used to determine mRNA for MCT-1 (Mm01315398_m1), SMCT-1 (Mm00520629_m1), Ffar2 (Mm02620654_s1), Ffar3 (Mm02621638_s1), PYY (Mm00520716_g1) and GLP-1 (Mm01269055_m1). mRNA levels were calculated relative to 36b4 (Mm00725448_s1) expression and normalized for expression levels of mice fed the control diet.

For immunoblot analysis, whole-cell lysate was prepared in lysis buffer from six mice per group and the protein concentrations were determined using the BCA Protein Assay kit (Pierce). Individual samples were mixed with loading buffer, heated for 5 min at 96°C and subjected to SDS-PAGE. Antibodies and their sources were AMP kinase (AMPK, no. 2532S; Cell Signaling) phosphorylated AMPK (pAMPK Thr172, no. 2531; Cell Signaling), acetyl CoA carboxylase (ACC, no. 3676P; Cell Signaling), phosphorylated ACC (pACC S79, no. 31931; Abcam), uncoupling protein 2 (UCP2, no. 6525; Santa Cruz), peroxisome proliferator-activated receptor γ (PPARγ, no. 2435; Cell Signaling) and fatty acid synthase (FASN, no. 3180; Cell Signaling). ß-actin (no. 2066; Sigma) was used as the loading control. Finally, horseradish peroxidase-conjugated anti-rabbit from donkey (Amersham Pharmacia Bioscience) or horseradish peroxidase-conjugated anti-goat from donkey (Dako, Glostrup, Denmark) and SuperSignal West Pico Chemiluminescent Substrate System (Pierce) were used. The immunoblots were analyzed by densitometry using Image Lab software (Bio-Rad).

### Statistics

All data are presented as mean values ± SEM. Statistical analysis was assessed by Mann-Whitney U-test or one-way ANOVA using the Tukey test for post-hoc analysis. Statistical significance was reached at a *p* value below 0.05.

## Results

### Guar gum protects against high-fat diet-induced obesity and insulin resistance

**Fig 1:**
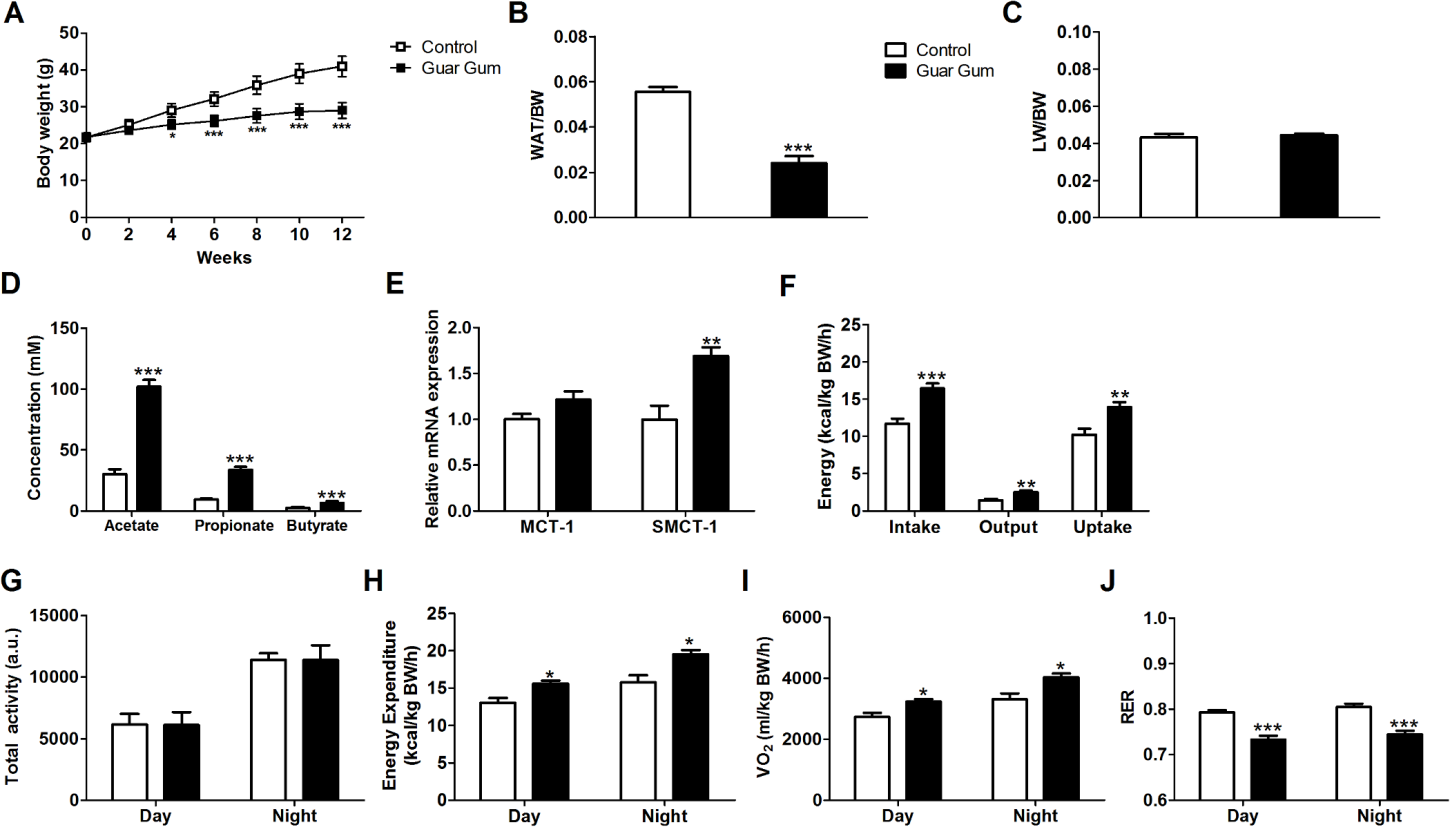
Guar gum protects against dietary-induced obesity. (A) Body weight evolution was monitored for 12 weeks. (B-C) White adipose tissue to body weight ratio and liver weight to body weight ratio after 12 weeks on diet. (D) Cecal SCFA concentrations were determined by GC/MS after 12 weeks on diet. (E) Cecal mRNA expression of genes involved in SCFA transport was assed via qPCR after 12 weeks on diet. (F) Energy balance was determined by measuring the energy content of the diet and dried homogenized feces after 10 weeks on diet. Uptake is defined as the difference between intake and output. (G-J) Total activity, energy expenditure, VO_2_ and RER were evaluated using indirect calorimetry data after 10 weeks on diet. Values are presented as mean ± SEM for n = 7–8; *p<0.05, ***p<0.001.

To examine the effects of guar gum during the development of the metabolic syndrome, C57Bl/6J mice were fed a semi-synthetic high-fat diet (HFD) supplemented with 0 or 10% guar gum for 12 weeks. As expected, HFD induced body weight (BW) gain, while guar gum supplementation attenuated the increase in BW (Fig 1A) and concomitantly reduced the mass of white adipose tissue (WAT) (Fig 1B). There was no difference in liver weight to body weight ratio between the groups (Fig 1C). Guar gum increased the cecal SCFA concentrations as well as the mRNA expression of the cecal SCFA transporter SMCT-1, while MCT-1 was not changed significantly (Fig 1D and 1E, respectively). To investigate the whole-body effect of guar gum on energy metabolism, the mice were subjected to indirect calorimetry. The reduced BW gain of guar gum-fed mice was not due to reduced food intake, as guar gum-fed mice had similar intake (S1 Fig). Actually, energy intake and uptake increased when normalized for BW (Fig 1F). Furthermore, guar gum did not affect the activity pattern of mice (Fig 1G), indicating that the lower BW on HFD was not due to different physical activity either. Rather, the phenotype was explained by enhanced energy expenditure (Fig 1H). Mice fed guar gum displayed a shift towards increased fatty acid oxidation, as indicated by higher O_2_ consumption rates and lower respiratory exchange ratio (RER) values (Fig 1I and 1J, respectively).

### Guar gum enhances oxidative metabolism via the same signaling cascade as SCFAs

In a previous study we demonstrated—by using selective PPARγ activators and repressors *in vitro* and organ-specific PPARγ knock-out mice *in vivo*—that adipose and hepatic PPARγ are critical mediators of the beneficial effects of SCFA on the metabolic syndrome, with clearly distinct and complementary roles [13]. Specifically, we showed that dietary SCFAs act through repression of PPARγ expression, subsequently increasing mitochondrial UCP2 expression and AMP/ATP ratio, leading to the activation of AMPK and culminating in enhanced oxidative metabolism in both liver and adipose tissue [13]. Here, we wondered if guar gum acts through the same signaling cascade as dietary SCFAs [13].

**Fig 2:**
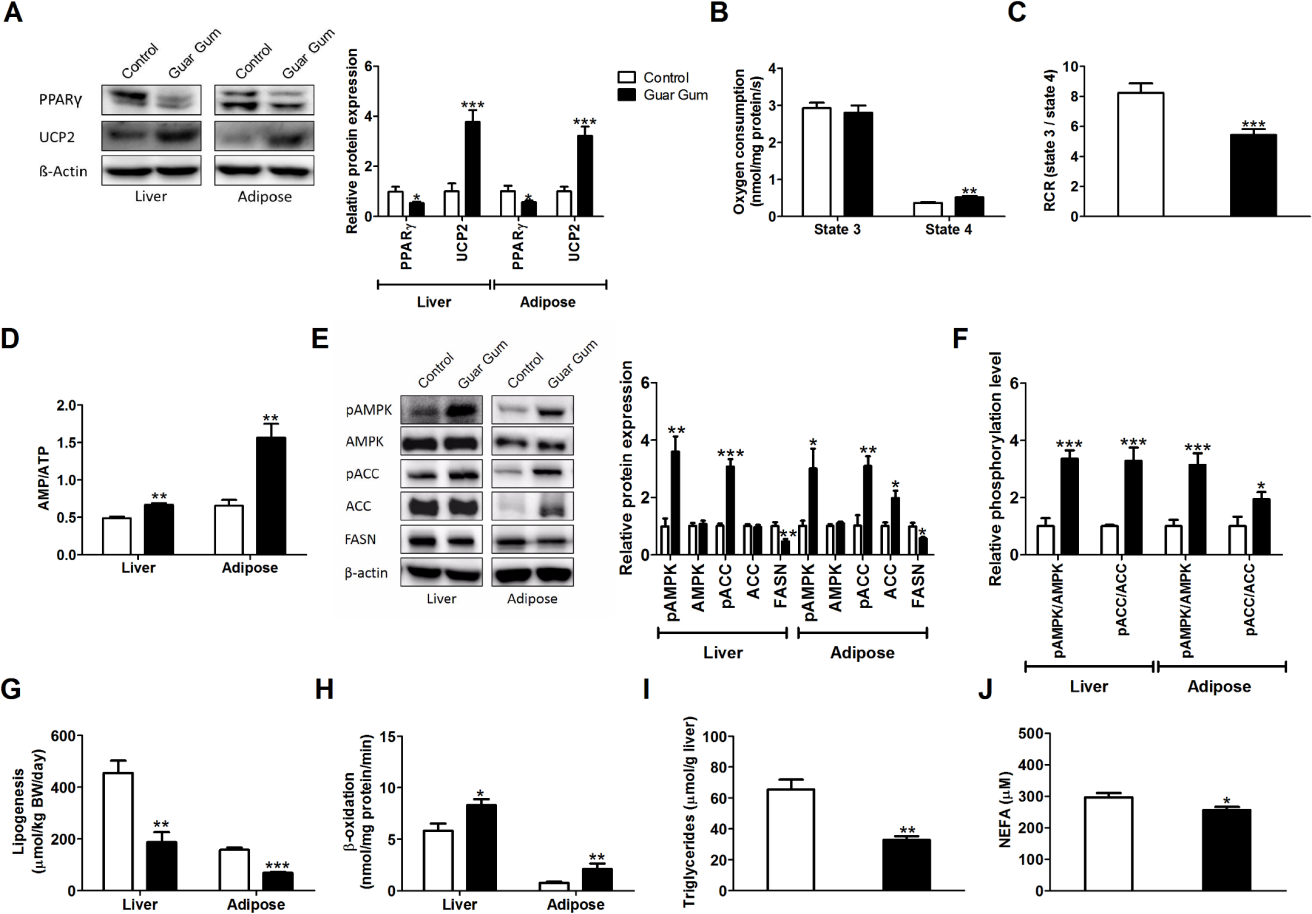
Guar gum decreases lipogenesis and increases mitochondrial fatty-acid oxidation in liver and adipose tissue. (A) PPARγ and UCP2 expression in liver and adipose tissue were analyzed by western blot of mice 12 weeks on diet. Quantification is shown in the upper panel. (B-C) After 12 weeks on diet, liver mitochondria were isolated and maximal ADP-stimulated oxygen consumption (*i*.*e*. state 3) and resting state oxygen consumption (*i*.*e*. state 4) were determined using palmitoyl CoA and malate as substrates. (D) AMP to ATP ratios in liver and adipose tissue were determined by HPLC after 12 weeks on diet. (E) pAMPK, AMPK, pACC, ACC and FASN expression in liver and adipose tissue were analyzed by western blot of mice 12 weeks on diet. Quantification is shown in the right panel. (F) Relative phosphorylation levels were calculated by the ratio of pAMPK/AMPK and pACC/ACC and normalized for control. (G-H) Lipogenesis and ß-oxidation in liver and adipose tissue after 12 weeks of diet. (I-J) Plasma NEFA concentrations and liver triglycerides after 12 weeks of diet. Values are presented as mean ± SEM for n = 6–8; *p<0.05, **p<0.01, ***p<0.001.

Indeed also guar gum supplementation decreased expression of PPARγ and increased the expression of UCP2 in both liver and adipose tissue (Fig 2A). UCP2 uncouples mitochondrial oxidative phosphorylation, and consistently guar gum caused a significant increase of the resting respiration rate (state 4) and a 1.5-fold decrease of the respiratory control ratio in isolated liver mitochondria (Fig 2B and 2C). Unfortunately, the low amount of mitochondria in WAT [25] precluded direct measurements of oxygen consumption in the latter tissue. Consistently with the increased mitochondrial uncoupling, the AMP/ATP ratio was increased in both liver and adipose tissue upon guar gum feeding (Fig 2D). The AMP/ATP ratio is a sensitive reflection of the metabolic state of the cell and a direct activator of AMPK [26]. Indeed, phosphorylation of AMPK and its downstream target acetyl-coenzyme A carboxylase (ACC) was increased after guar gum-feeding (Fig 2E). Total AMPK expression was not affected in liver and adipose tissue, whereas total ACC levels were similar in liver tissue but increased in adipose tissue in guar gum-fed mice (Fig 2E). However, in both liver and adipose tissue the pAMPK/AMPK and pACC/ACC ratio were significantly increased upon guar gum feeding (Fig 2F). Increased phosphorylation of AMPK indirectly decreases expression of fatty acid synthase (FASN) [27,28], which we also observed in the guar gum-supplemented mice (Fig 2E). Phosphorylation of ACC inactivates the enzyme and thereby decreases the concentration of its product malonyl-CoA. Malonyl-CoA is a substrate for fatty-acid synthesis and an inhibitor of carnitine-palmitoyl transferase 1, a main controlling enzyme of miotochondrial fatty-acid β-oxidation. Together, this resulted in a decrease in lipogenesis and increase in lipid oxidative capacity in both liver and adipose tissue in guar gum-fed mice (Fig 2G and 2H). Consequently, hepatic triglycerides and plasma NEFA concentrations decreased upon guar gum feeding (Fig 2I and 2J).

In conclusion, dietary guar gum induced the same signaling cascade in liver and adipose tissue as recently described for dietary SCFAs [13], as should be expected if SCFAs are the main molecular mediators of the guar gum-induced effects.

### Guar gum improves peripheral glucose and insulin handling

**Fig 3:**
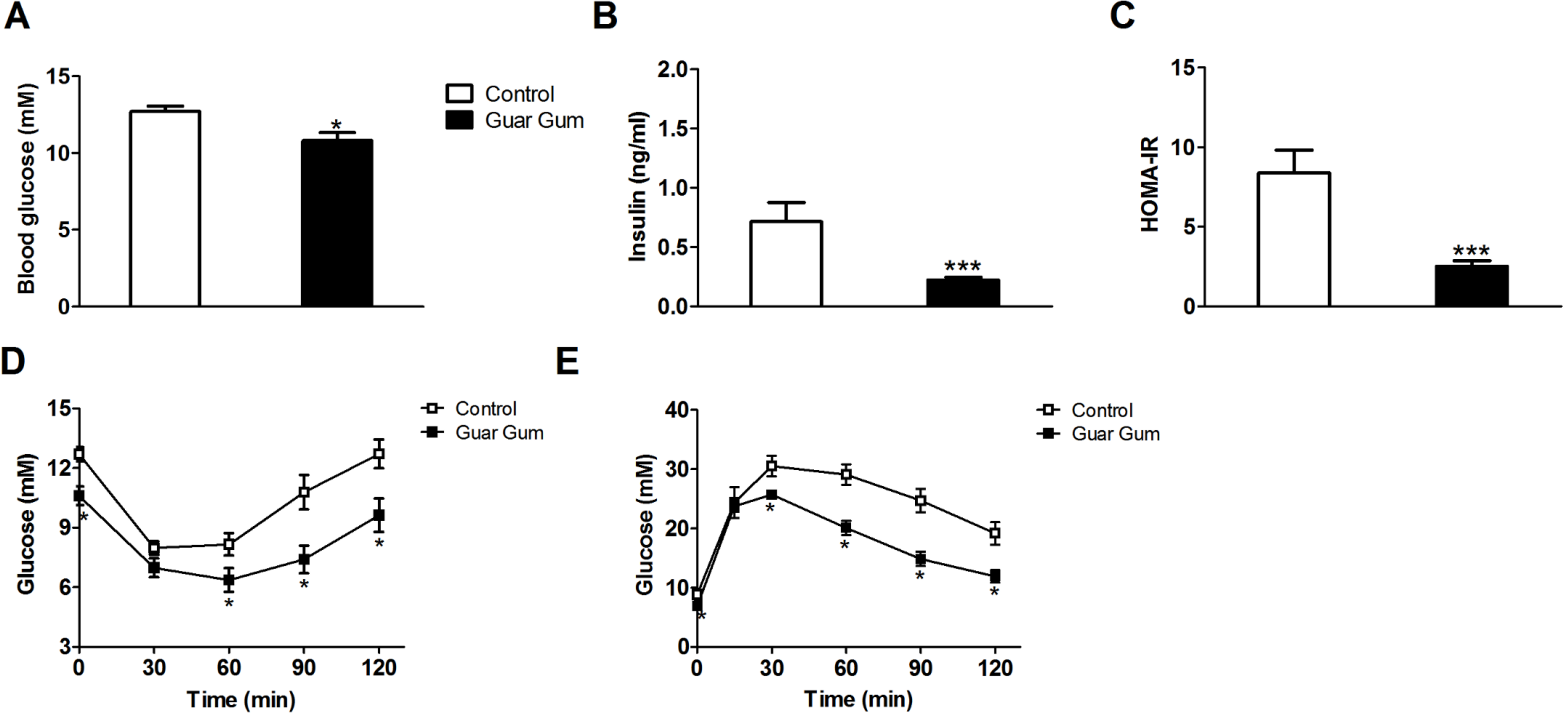
Guar gum increases insulin and glucose sensitivity. (A-B) Blood glucose and insulin concentrations upon a 4h fast after 12 weeks of diet. (C) HOMA-IR was calculated from fasted glucose and insulin levels after 12 weeks on diet. (D) Insulin tolerance tests were performed on mice for 11 weeks on their respective diets upon a 4h fast. (E) After 11 weeks on their respective diets an intraperitoneal glucose tolerance test was performed in mice that were fasted overnight for 9h. Values are presented as mean ± SEM for n = 6–8; *p<0.05, ***p<0.001.

**Fig 4:**
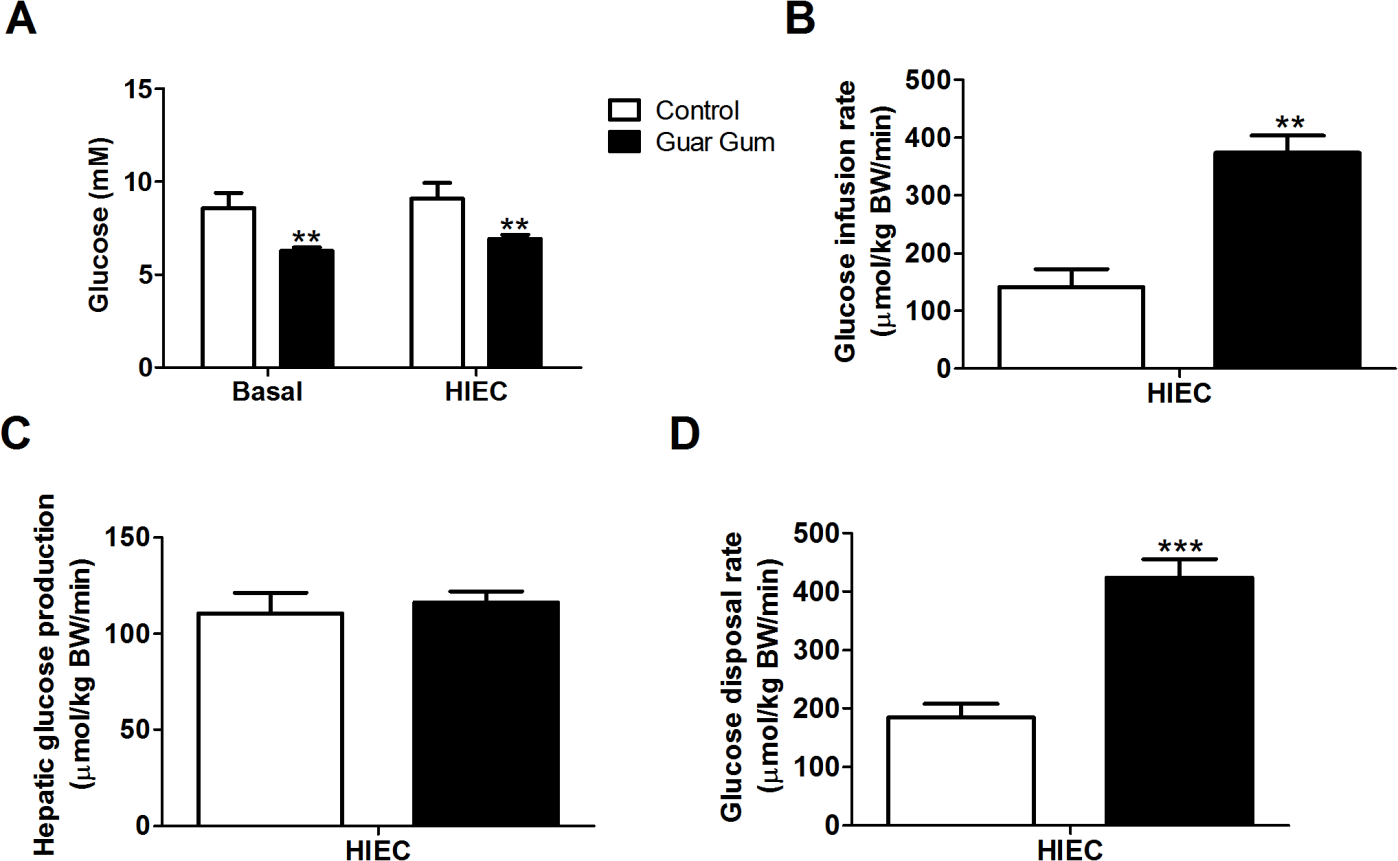
Guar gum enhances peripheral glucose handling. (A) Average blood glucose concentrations during basal and hyperinsulinemic-euglycemic clamps (HIEC) conditions after 12 weeks of diet. (B) Average glucose infusion rates needed to maintain euglycemic conditions. (C-D) Hepatic glucose production and peripheral glucose disposal rate during HIEC conditions. Values are presented as mean ± SEM for n = 6–8; *p<0.05, **p<0.01, ***p<0.001 Guar gum vs. control.

In agreement with our previous results [10], fasting plasma glucose and insulin concentrations, and thereby HOMA-IR levels, were significantly lower in guar gum-supplemented mice (Fig 3A–3C). Glucose disposal upon insulin delivery was slightly increased and glucose tolerance was markedly enhanced in guar gum-fed mice (Fig 3D and 3E). To further investigate the improved glucose and insulin handling, hyperinsulinemic-euglycemic clamp (HIEC) studies under matched insulin exposure were conducted as described previously [19]. Under basal conditions, guar gum supplementation resulted in decreased glucose levels (Fig 4A). During hyperinsulinemic conditions, the glucose-infusion rate was adjusted to keep the same euglycemic state as under basal conditions (Fig 4A). The glucose-infusion rate required for maintaining euglycemia (a measure of whole-body insulin sensitivity) was approximately 2.5-fold higher in guar gum-supplemented mice compared to control mice (Fig 4B). Although there was no significant difference in the hepatic glucose production rate during the HIEC (Fig 4C), the degree to which insulin stimulated the rate of glucose uptake by peripheral tissues (primarily muscle and adipose tissue) was significantly elevated in guar gum-fed mice (Fig 4D). These data were normalized for BW. Without BW correction, glucose-infusion rate and glucose uptake remained significantly increased in guar gum-fed mice whereas hepatic glucose production was significantly decreased compared to control-fed mice (S2A–S2C Fig), suggesting that increased hepatic insulin sensitivity may also contribute to improved glucose handling.

### Guar gum and cecal SCFAs increase plasma GLP-1 concentrations

**Fig 5:**
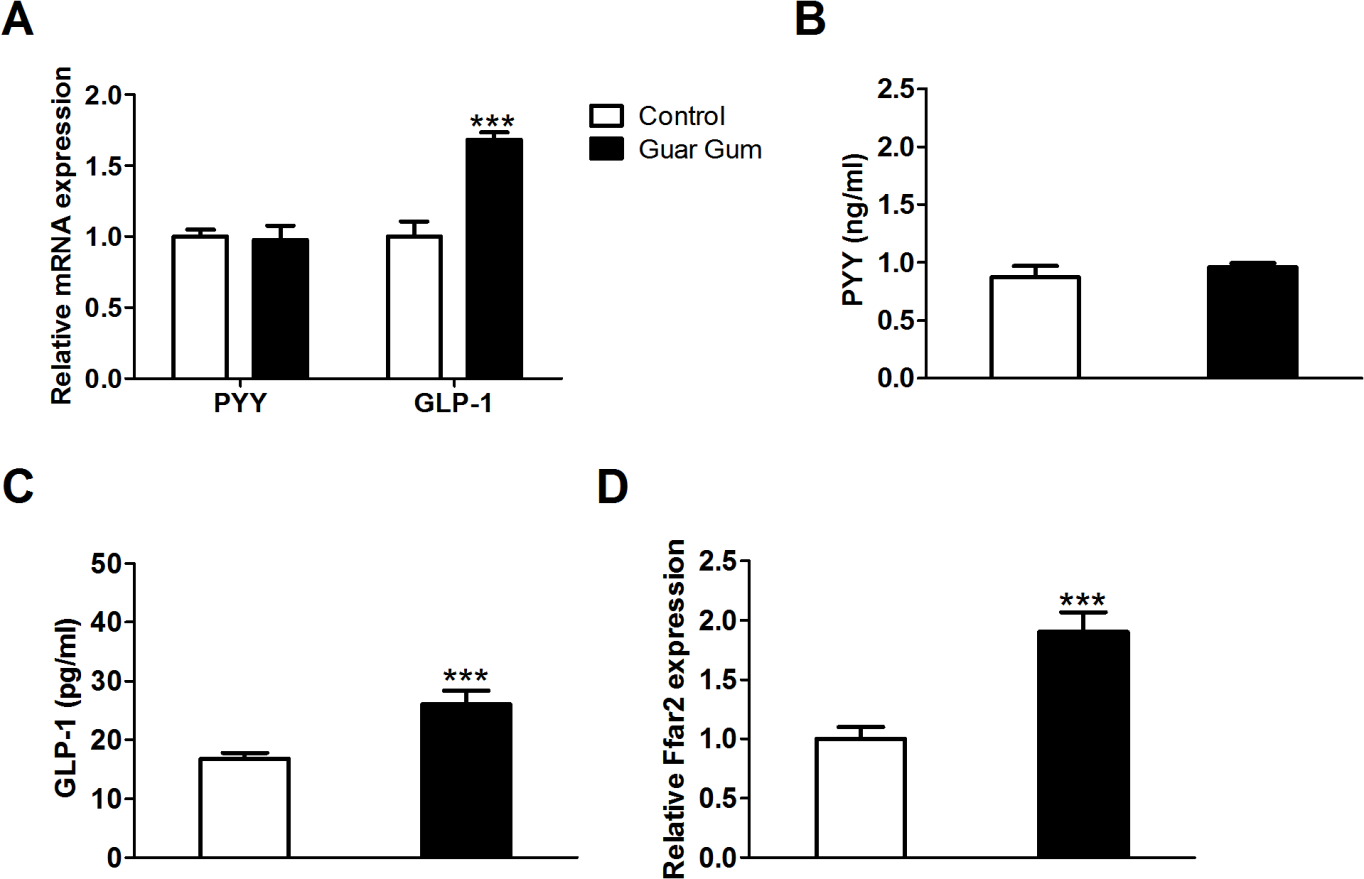
Guar gum increases colonic GLP-1 expression. (A) Cecal mRNA expression of PYY and GLP-1 were assessed by qPCR after 12 weeks of diet. (B-C) Plasma PYY and GLP-1 concentrations after 12 weeks of diet. (D) Cecal mRNA expression of Ffar2 was assessed by qPCR after 12 weeks of diet. Values are presented as mean ± SEM for n = 6–8; ***p<0.001.

In contrast to dietary SCFA-supplementation [13], guar gum did not only enhance insulin-stimulated glucose metabolism, but it also decreased basal plasma glucose concentrations and enhanced glucose tolerance (Figs 3 and 4). Together this suggests that there might be an additional factor, besided PPARγ repression, that improved glucose metabolism in guar gum-fed mice. The colonic hormones peptide YY (PYY) and glucagon-like peptide-1 (GLP-1) both regulate peripheral glucose metabolism [29,30]. PYY reinforces the insulin action on glucose disposal in muscle and adipose tissue [29] and GLP-1 increases peripheral glucose-mediated glucose uptake independently of hyperinsulinemia [30,31]. Guar gum feeding increased the cecal mRNA expression and plasma concentration of GLP-1, whereas no effect was observed on PYY (Fig 5A–5C).

**Fig 6:**
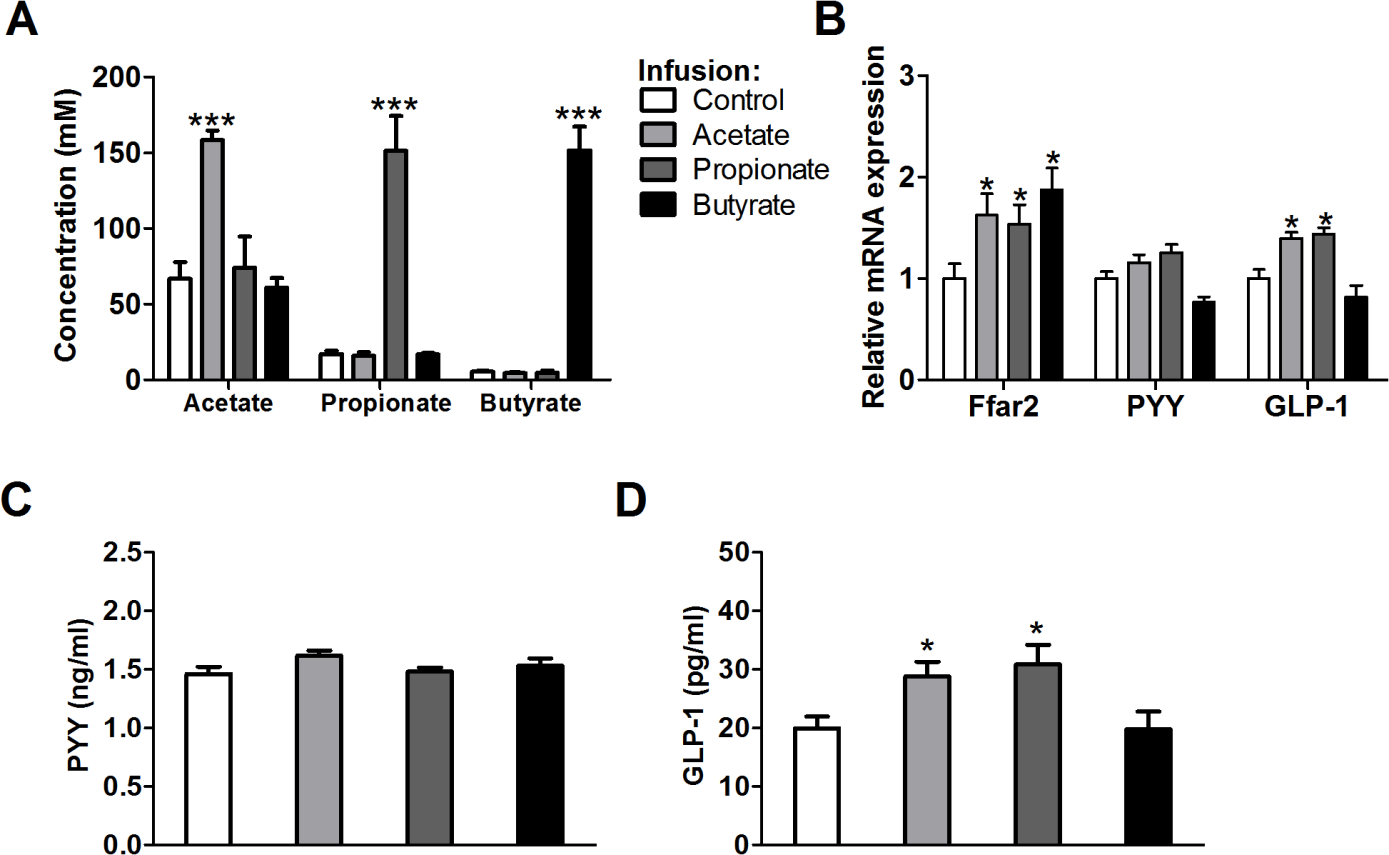
Cecal acetate and propionate increase GLP-1 expression. (A) Cecal SCFA concentrations after 6h cecal SCFA infusion in mice fed HFD without guar gum for 6 weeks. (B) After 6h cecal SCFA infusion cecal mRNA expression of Ffar2, PYY and GLP-1 were assessed qPCR. (C-D) Plasma PYY and GLP-1 concentrations after 6h cecal SCFA infusion. Values are presented as mean ± SEM for n = 6–8; *p<0.05, ***p<0.001.

Colonic SCFAs stimulate GLP-1 secretion and mice lacking the free fatty acid receptor (Ffar) 2 showed reduced SCFA-triggered GLP-1 secretion and a parallel impairment of glucose tolerance [32,33]. Guar gum also increased cecal mRNA expression of the SCFA receptor Ffar2 (Fig 5D). Therefore, we wondered if cecally supplemented SCFAs were capable of inducing GLP-1 expression. To investigate this, we infused each individual SCFA directly into the cecum of conscious, unrestrained mice that were fed the HFD without guar gum. We infused each SCFA at the same rate and this rate was based on the recommended intake of dietary fiber for humans (see Materials and Methods). Infusing SCFAs resulted in an increase in cecal concentration of the infused SCFA without altering the concentrations of the other SCFAs (Fig 6A). All three SCFAs increased the cecal mRNA expression of Ffar2 (Fig 6B). However, only acetate and propionate infusion increased cecal GLP-1 mRNA expression and plasma concentration, whereas cecal PYY mRNA expression and plasma concentration did not respond to any of the three SCFA infusions (Fig 6B–6D). Together, these data suggest that guar gum increases peripheral glucose disposal at least partially by enhancing GLP-1 secretion, mediated through the cecal or colonic SCFAs acetate and propionate.

## Discussion

We demonstrated that guar gum protects against HFD-induced obesity and insulin resistance through the same signaling cascade in liver and adipose tissue as supplemented SCFAs. In addition, guar gum exerts improved peripheral glucose handling, which is at least partially mediated by the SCFA-induced colonic hormone GLP-1.

The anti-obesogenic effect of guar gum observed in the present study is in agreement with earlier studies that showed that guar gum can prevent and reverse body weight gain in rodents and humans [6,7,34]. Recently, we showed that the dose-dependent amelioration of the metabolic syndrome by guar gum correlates with *in vivo* SCFA uptake fluxes by the host, suggesting that the physiological effects of guar gum are primarily mediated by SCFAs that are taken up by the host [10]. In addition, we showed that SCFAs protect against the metabolic syndrome via a signaling cascade that involves PPARγ repression and AMPK activation [13]. Here we demonstrate that guar gum supplementation works in the same way as SCFAs in the protection against HFD-induced obesity and insulin resistance, namely by repressing PPARγ expression, subsequently increasing mitochondrial UCP2 expression and AMP/ATP ratio, leading to the activation of AMPK and culminating in enhanced fatty-acid oxidation in both liver and adipose tissue. We note that—unlike corn starch–guar gum is only taken up after bacterial fermentation. Acetic acid, the most abundant bacterial product, however, has the same C:H:O ratio as carbohydrates and its oxidation, therefore, results also in the same respiratory exchange ratio. Thus, the decreased RER in the guar-gum fed mice is most likely due to genuine long-chain fatty-acid oxidation. The fact that guar gum elicits the same molecular and physiological response as SCFA, further corroborates the notion that absorbed SCFAs are the main molecular mediators of the beneficial effects of guar gum on the metabolic syndrome.

Guar gum supplementation decreases fasting plasma glucose levels in healthy, type 1 and type 2 diabetic humans but a clear mechanism has been lacking so far [4,5,35,36]. Here we demonstrate that guar gum decreases plasma glucose levels by increasing the rate of glucose clearance by peripheral tissues. Dietary SCFA supplementation elicited no effect on basal plasma glucose levels and glucose tolerance [13], while we show here that guar gum has an effect on glucose metabolism. This may be explained by the fact that SCFAs produced by cecal bacterial fermentation of dietary fibers appear in the cecum, while orally ingested SCFAs are absorbed in the small intestine and do not reach the cecum (S3A and S3B Fig). This is likely to affect their metabolic effects, since different hormonal and regulatory responses are triggered in the different places in the gastrointestinal tract [37]. Consistently, we found increased plasma levels of GLP-1 in mice supplemented with guar gum or cecally infused acetate or propionate (Figs 5 and 6), but not with orally ingested SCFAs (S3C Fig). GLP-1 has been shown to increase peripheral glucose metabolism independently of hyperinsulinemia [38–40], which is consistent with our results that show that guar gum mainly acts on peripheral tissues and that orally ingested SCFAs did not affect basal glucose metabolism [13]. In addition, we show that unlike acetate and propionate, butyrate does not induce GLP-1 expression when infused directly into the cecum. This selective effect on GLP-1 is in agreement with the result that SCFAs increase GLP-1 through Ffar2 activation [32], which has a preference for acetate and propionate [41,42].

Apart from guar gum there are many other different dietary fibers that elicit benificial effects in healthy and metabolic syndrome patients [43–45]. As most of these fibers also increase cecal or fecal SCFAs [9,43], we speculate that their effects are mediated by SCFAs as well. But, since cecal and fecal SCFA concentrations do not necessarily reflect their uptake rates [10], it is difficult to compare these studies directly. Along similar lines, we conclude here that the guar gum-induced effects are primarily mediated by the SCFAs, but it is possible that additional metabolites are involved. The gut microbiota produces a wide range of other metabolites that have potential biological functions in host energy metabolism [46]. Various dietary modulations alter the composition of the gut microbiota and, subsequently, the host metabolic phenotype and disease risk [47,48].

Overall, this study provides novel molecular insights into the beneficial effects of guar gum on the metabolic syndrome and strengthens the potential role of guar gum as a dietary-fiber intervention.

## Supporting Information

S1 File Animals and Experimental Design and Supplemental References (DOCX)

S1 Fig Energy balance per mouse. (DOCX)

S2 Fig Hyperinsulinemic-euglycemic clamps results per mouse. (DOCX)

S3 Fig Dietary SCFA-supplementation does not affect GLP-1 expression. (DOCX)

## References

[1] Galisteo M, Duarte J, Zarzuelo A. Effects of dietary fibers on disturbances clustered in the metabolic syndrome. J Nutr Biochem. 2008;19: 71–84. 17618108 10.1016/j.jnutbio.2007.02.009

[2] Misra A, Singhal N, Khurana L. Obesity, the Metabolic Syndrome, and Type 2 Diabetes in Developing Countries: Role of Dietary Fats and Oils. J Am Coll Nutr. 2010;29: 289S–301S. 20823489 10.1080/07315724.2010.10719844

[3] Papathanasopoulos A, Camilleri M. Dietary Fiber Supplements: Effects in Obesity and Metabolic Syndrome and Relationship to Gastrointestinal Functions. Gastroenterology. 2010;138: 65–72. 10.1053/j.gastro.2009.11.045 19931537 PMC2903728

[4] Landin K, Holm G, Tengborn L, Smith U. Guar gum improves insulin sensitivity, blood lipids, blood pressure, and fibrinolysis in healthy men. Am J Clin Nutr. 1992;56: 1061–1065. 1442658 10.1093/ajcn/56.6.1061

[5] Dall'alba V, Silva FM, Antonio JP, Steemburgo T, Royer CP, Almeida JC, et al Improvement of the metabolic syndrome profile by soluble fibre—guar gum—in patients with type 2 diabetes a randomised clinical trial. Br J Nutr. 2013;110: 1601–1610. 10.1017/S0007114513001025 23551992

[6] Cicero AFG, Derosa G, Bove M, Imola F, Borghi C, Gaddi AV. Psyllium improves dyslipidaemia, hyperglycaemia and hypertension, while guar gum reduces body weight more rapidly in patients affected by metabolic syndrome following an AHA Step 2 diet. Mediterr J Nutr Metab. 2010;3: 47–57.

[7] Krotkiewski M. Effect of guar gum on body-weight, hunger ratings and metabolism in obese subjects. Br J Nutr. 1984;52: 97–105. 6331498 10.1079/bjn19840075

[8] Flint HJ, Scott KP, Louis P, Duncan SH. The role of the gut microbiota in nutrition and health. Nat Rev Gastroenterol Hepatol. 2012;9: 577–589. 10.1038/nrgastro.2012.156 22945443

[9] den Besten G, van Eunen K, Groen AK, Venema K, Reijngoud D, Bakker BM. The role of short-chain fatty acids in the interplay between diet, gut microbiota and host energy metabolism. J Lipid Res. 2013;54: 2325–2340. 10.1194/jlr.R036012 23821742 PMC3735932

[10] den Besten G, Havinga R, Bleeker A, Rao S, Gerding A, van Eunen K, et al The Short-Chain Fatty Acid Uptake Fluxes by Mice on a Guar Gum Supplemented Diet Associate with Amelioration of Major Biomarkers of the Metabolic Syndrome. PLoS ONE. 2014;9: e107392 10.1371/journal.pone.0107392 25203112 PMC4159349

[11] Gao Z, Yin J, Zhang J, Ward RE, Martin RJ, Lefevre M, et al Butyrate Improves Insulin Sensitivity and Increases Energy Expenditure in Mice. Diabetes. 2009;58: 1509–1517. 10.2337/db08-1637 19366864 PMC2699871

[12] Lin HV, Frassetto A, Kowalik EJJ, Nawrocki AR, Lu MM, Kosinski JR, et al Butyrate and Propionate Protect against Diet-Induced Obesity and Regulate Gut Hormones via Free Fatty Acid Receptor 3-Independent Mechanisms. PLoS ONE. 2012;7: e35240 10.1371/journal.pone.0035240 22506074 PMC3323649

[13] den Besten G, Bleeker A, Gerding A, van Eunen K, Havinga R, van Dijk TH, et al Short-Chain Fatty Acids protect against High-Fat Diet-Induced Obesity via a PPARγ-dependent switch from lipogenesis to fat oxidation. Diabetes. 2015;64: 2398–2408. 10.2337/db14-1213 25695945

[14] Bligh EG, Dyer WJ. A rapid method of total lipid extraction and purification. Can J Biochem Physiol. 1959;37: 911–917. 13671378 10.1139/o59-099

[15] Oosterveer MH, van Dijk TH, Tietge UJ, Boer T, Havinga R, Stellaard F, et al High Fat Feeding Induces Hepatic Fatty Acid Elongation in Mice. PLoS One. 2009;4: e6066 10.1371/journal.pone.0006066 19557132 PMC2699051

[16] Hirschey MD, Shimazu T, Goetzman E, Jing E, Schwer B, Lombard DB, et al SIRT3 regulates fatty acid oxidation via reversible enzyme deacetylation. Nature. 2010;464: 121–125. 10.1038/nature08778 20203611 PMC2841477

[17] Miller RA, Chu Q, Xie J, Foretz M, Viollet B, Birnbaum MJ. Biguanides suppress hepatic glucagon signalling by decreasing production of cyclic AMP. Nature. 2013;494: 256–260. 10.1038/nature11808 23292513 PMC3573218

[18] Kuznetsov AV, Lassnig B, Stadlmann S, Rieger G, Gnaiger E. Selected media and chemicals for respirometry with mitochondria and permeabilized cells. OROBOROS Bioenerg News. 1998;3: 1–9.

[19] Grefhorst A, van Dijk TH, Hammer A, van der Sluijs FH, Havinga R, Havekes LM, et al Differential effects of pharmacological liver X receptor activation on hepatic and peripheral insulin sensitivity in lean and ob/ob mice. Am J Physiol Endocrinol Metab. 2005;289: E829–E838. 15941783 10.1152/ajpendo.00165.2005

[20] Mildaziene V, Nauciene Z, Baniene R, Grigiene J. Multiple Effects of 2,2`,5,5`-Tetrachlorobiphenyl on Oxidative Phosphorylation in Rat Liver Mitochondria. Toxicol Sci. 2002;65: 220–227. 11812926 10.1093/toxsci/65.2.220

[21] Gnaiger E, Kuznetsov AV, Schneeberger S, Seiler R, Brandacher G, Steurer W, et al Mitochondria in the cold In: Heldmaier G, Klingenspor M, editors. Life in the Cold.: Springer; 2000 pp. 431–442.

[22] den Besten G, Lange K, Havinga R, van Dijk TH, Gerding A, van Eunen K, et al Gut-derived short-chain fatty acids are vividly assimilated into host carbohydrates and lipids. Am J Physiol Gastrointest Liver Physiol. 2013;305: G900–910. 10.1152/ajpgi.00265.2013 24136789

[23] National Academy of Sciences. Dietary, functional, and total fiber In: Anonymous Dietary Reference Intakes for Energy, Carbohydrate, Fiber, Fat, Fatty Acids, Cholesterol, Protein, and Amino Acids (Macronutrients). Washington, D.C.: National Academies Press; 2005 pp. 339–400.

[24] Bergman EN. Energy contributions of volatile fatty acids from the gastrointestinal tract in various species. Physiol Rev. 1990;70: 567–590. 2181501 10.1152/physrev.1990.70.2.567

[25] Cannon B, Nedergaard J. Studies of thermogenesis and mitochondrial function in adipose tissues. Methods Mol Biol. 2008;456: 109–121. 10.1007/978-1-59745-245-8_8 18516556

[26] Carling D, Mayer FV, Sanders MJ, Gamblin SJ. AMP-activated protein kinase: nature's energy sensor. Nat Chem Biol. 2011;7: 512–518. 10.1038/nchembio.610 21769098

[27] Foretz M, Carling D, Guichard C, Ferré P, Foufelle F. AMP-activated protein kinase inhibits the glucose-activated expression of fatty acid synthase gene in rat hepatocytes. J Biol Chem. 1988;273: 14767–14771. 10.1074/jbc.273.24.14767 9614076

[28] Leclerc I, Kahn A, Doiron B. The 5'-AMP-activated protein kinase inhibits the transcriptional stimulation by glucose in liver cells, acting through the glucose response complex. FEBS Lett. 1998;431: 180–184. 9708898 10.1016/s0014-5793(98)00745-5

[29] van den Hoek AM, Heijboer AC, Corssmit EPM, Voshol PJ, Romijn JA, Havekes LM, et al PYY3–36 Reinforces Insulin Action on Glucose Disposal in Mice Fed a High-Fat Diet. Diabetes. 2004;53: 1949–1952. 15277371 10.2337/diabetes.53.8.1949

[30] Barrera JG, Sandoval DA, D'Alessio DA, Seele RJ. GLP-1 and energy balance: an integrated model of short-term and long-term control. Nat Rev Endocrinol. 2011;7: 507–516. 10.1038/nrendo.2011.77 21647189 PMC4231434

[31] Meier JJ. GLP-1 receptor agonists for individualized treatment of type 2 diabetes mellitus. Nat Rev Endocrinol. 2012;8: 728–742. 10.1038/nrendo.2012.140 22945360

[32] Tolhurst G, Heffron H, Lam YS, Parker HE, Habib AM, Diakogiannaki E, et al Short-Chain Fatty Acids Stimulate Glucagon-Like Peptide-1 Secretion via the G-Protein–Coupled Receptor FFAR2. Diabetes. 2011;61: 364–371. 10.2337/db11-1019 22190648 PMC3266401

[33] Freeland KR, Wolever TM. Acute effects of intravenous and rectal acetate on glucagon-like peptide-1, peptide YY, ghrelin, adiponectin and tumour necrosis factor-alpha. Br J Nutr. 2010;103: 460–466. 10.1017/S0007114509991863 19818198

[34] Uusitupa M, Siitonen O, Savolainen K, Silvasti M, Penttilä I, Parviainen M. Metabolic and nutritional effects of long-term use of guar gum in the treatment of noninsulin-dependent diabetes of poor metabolic control. Am J Clin Nutr. 1989;49: 345–351. 2537003 10.1093/ajcn/49.2.345

[35] Smith U, Holm G. Effect of a modified guar gum preparation on glucose and lipid levels in diabetics and healthy volunteers. Atherosclerosis. 1982;45: 1–10. 6297515 10.1016/0021-9150(82)90166-6

[36] Ebeling P, Yki-Järvinen H, Aro A, Helve E, Sinisalo M, Koivisto VA. Glucose and lipid metabolism and insulin sensitivity in type 1 diabetes the effect of guar gum. Am J Clin Nutr. 1988;48: 98–103. 3291601 10.1093/ajcn/48.1.98

[37] Murphy KG, Bloom SR. Gut hormones and the regulation of energy homeostasis. Nature. 2006;444: 854–859. 17167473 10.1038/nature05484

[38] D'Alessio DA, Kahn SE, Leusner CR, Ensinck JW. Glucagon-like peptide 1 enhances glucose tolerance both by stimulation of insulin release and by increasing insulin-independent glucose disposal. J Clin Invest. 1994;93: 2263–2266. 8182159 10.1172/JCI117225 PMC294382

[39] Burcelin R, Da Costa A, Drucker D, Thorens B. Glucose competence of the hepatoportal vein sensor requires the presence of an activated glucagon-like peptide-1 receptor. Diabetes. 2001;50: 1720–1728. 11473030 10.2337/diabetes.50.8.1720

[40] Ionut V, Hucking K, Liberty IF, Bergman RN. Synergistic effect of portal glucose and glucagon-like peptide-1 to lower systemic glucose and stimulate counter-regulatory hormones. Diabetologia. 2005;48: 967–975. 15830188 10.1007/s00125-005-1709-3

[41] Brown AJ, Goldsworthy SM, Barnes AA, Eilert MM, Tcheang L, Daniels D, et al The Orphan G Protein-coupled Receptors GPR41 and GPR43 Are Activated by Propionate and Other Short Chain Carboxylic Acids. J Biol Chem. 2003;278: 11312–11319. 12496283 10.1074/jbc.M211609200

[42] Le Poul E, Loison C, Struyf S, Springael JY, Lannoy V, Decobecq ME, et al Functional Characterization of Human Receptors for Short Chain Fatty Acids and Their Role in Polymorphonuclear Cell Activation. J Biol Chem. 2003;278: 25481–25489. 12711604 10.1074/jbc.M301403200

[43] Topping DL, Clifton PM. Short-Chain Fatty Acids and Human Colonic Function: Roles of Resistant Starch and Nonstarch Polysaccharides. Physiol Rev. 2001;81: 1031–1064. 11427691 10.1152/physrev.2001.81.3.1031

[44] Galisteo M, Duarte J, Zarzuelo A. Effects of dietary fibers on disturbances clustered in the metabolic syndrome. J Nutr Biochem. 2008;19: 71–84. 17618108 10.1016/j.jnutbio.2007.02.009

[45] Bingham SA, Day NE, Luben R, Ferrari P, Slimani N, Norat T, et al Dietary fibre in food and protection against colorectal cancer in the European Prospective Investigation into Cancer and Nutrition (EPIC): an observational study. The Lancet. 2003;361: 1496–1501. 10.1016/s0140-6736(03)13174-1 12737858

[46] Nicholson JK, Holmes E, Kinross J, Burcelin R, Gibson G, Jia W, et al Host-Gut Microbiota Metabolic Interactions. Science. 2012;336: 1262–1267. 10.1126/science.1223813 22674330

[47] Holmes E, Li JV, Marchesi JR, Nicholson JK. Gut microbiota composition and activity in relation to host metabolic phenotype and disease risk. Cell Metab. 2012;16: 559–564. 10.1016/j.cmet.2012.10.007 23140640

[48] Claus SP, Ellero SL, Berger B, Krause L, Bruttin A, Molina J, et al Colonization-Induced Host-Gut Microbial Metabolic Interaction. MBio. 2011;2: e00271–e00281. 10.1128/mBio.00271-10 21363910 PMC3045766

