# Supplementary material for: Protection against the Metabolic Syndrome by Guar Gum-Derived Short-Chain Fatty Acids Depends on Peroxisome Proliferator-Activated Receptor γ and Glucagon-Like Peptide-1
Source: PLoS One. 2015 Aug 20;10(8):e0136364. doi: 10.1371/journal.pone.0136364 (PMC4546369; doi:10.1371/journal.pone.0136364)
Supplement: S1 File — (DOCX) [file pone.0136364.s001.docx]

**Animals and Experimental Design**

Male C57Bl/6J mice (Charles River, L’Arbresle Cedex, France), 2 months of age, were housed in a light- and temperature-controlled facility (lights on from 6:30 a.m. to 6:30 p.m., 21 °C) with free access to water and food. The experimental groups were fed a high-fat semi-synthetic diet (D12451, Research Diet Services, Wijk Bij Duurstede, The Netherlands) in which 45% of calories were from palm oil fat. For the SCFA diets, sodium acetate (S2889; Sigma), sodium propionate (P1880; Sigma) or sodium butyrate (303410; Sigma) was incorporated into the diet at 5% (w/w) as described previously [1].

Cecal SCFA concentrations, mRNA expression and plasma GLP-1 concentration was determined as described in the Materials and Methods.

**References**

1. den Besten G, Bleeker A, Gerding A, van Eunen K, Havinga R, van Dijk TH, et al. Short-Chain Fatty Acids protect against High-Fat Diet-Induced Obesity via a PPARγ-dependent switch from lipogenesis to fat oxidation. Diabetes. 2015;64: 2398-2408.
