# Supplementary figures and images for: Protection against the Metabolic Syndrome by Guar Gum-Derived Short-Chain Fatty Acids Depends on Peroxisome Proliferator-Activated Receptor γ and Glucagon-Like Peptide-1
Source: PLoS One. 2015 Aug 20;10(8):e0136364. doi: 10.1371/journal.pone.0136364 (PMC4546369; doi:10.1371/journal.pone.0136364)

**S1 Fig. Energy balance per mouse.** Values are presented as mean ± SEM for n=7-8


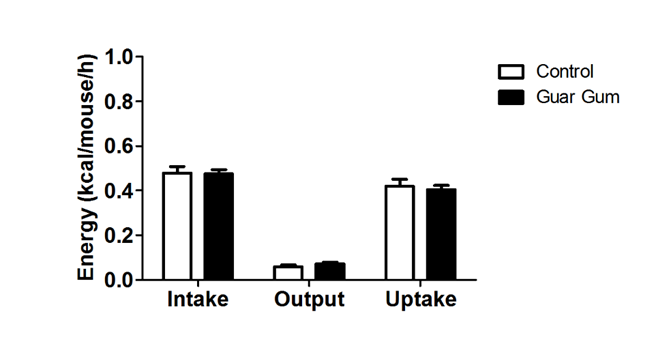

Supplement: S1 Fig — (DOCX) [file pone.0136364.s002.docx]
