# Supplementary material for: Protection against the Metabolic Syndrome by Guar Gum-Derived Short-Chain Fatty Acids Depends on Peroxisome Proliferator-Activated Receptor γ and Glucagon-Like Peptide-1
Source: PLoS One. 2015 Aug 20;10(8):e0136364. doi: 10.1371/journal.pone.0136364 (PMC4546369; doi:10.1371/journal.pone.0136364)

**S2 Fig. Hyperinsulinemic-euglycemic clamps results per mouse.** (A) Average glucose infusion rates needed to maintain euglycemic conditions. (B-C) Hepatic glucose production and peripheral glucose disposal rate during HIEC conditions. Values are presented as mean ± SEM for n=6-8; *p<0.05, **p<0.01 Guar gum vs. Control.


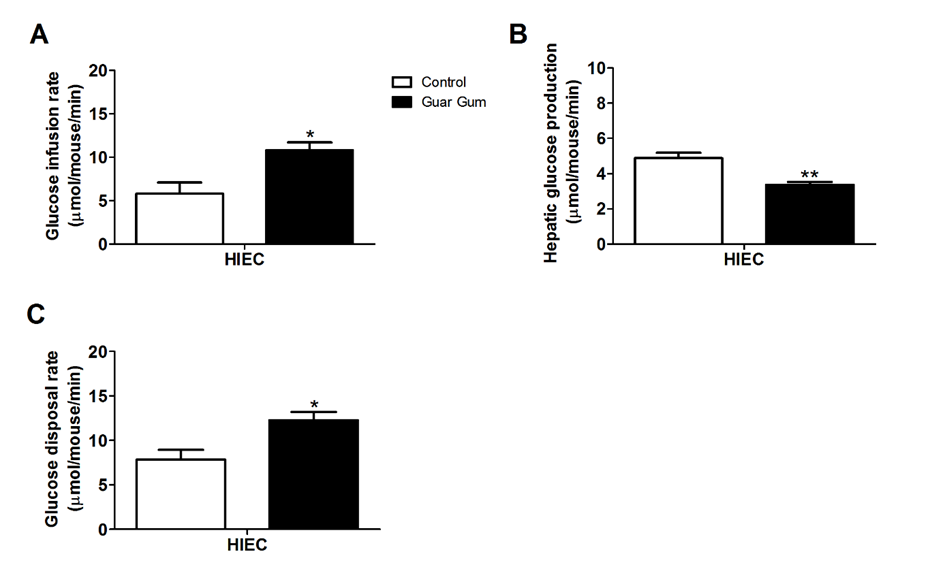

Supplement: S2 Fig — (DOCX) [file pone.0136364.s003.docx]
