# Supplementary material for: Protection against the Metabolic Syndrome by Guar Gum-Derived Short-Chain Fatty Acids Depends on Peroxisome Proliferator-Activated Receptor γ and Glucagon-Like Peptide-1
Source: PLoS One. 2015 Aug 20;10(8):e0136364. doi: 10.1371/journal.pone.0136364 (PMC4546369; doi:10.1371/journal.pone.0136364)

**S3 Fig. Dietary SCFA-supplementation does not affect GLP-1 expression.** (A) Cecal SCFA concentrations after 12 weeks of SCFA feeding (S1 File). (B) Cecal mRNA expression of genes involved in SCFA transport (MCT-1 and SMCT-1), SCFA signaling (Ffar2 and Ffar3) and PYY and GLP-1 were assed via qPCR after 12 weeks of SCFA feeding. (C) Plasma GLP-1 concentration after 12 weeks of SCFA feeding. Values are presented as mean ± SEM for n=6-8.


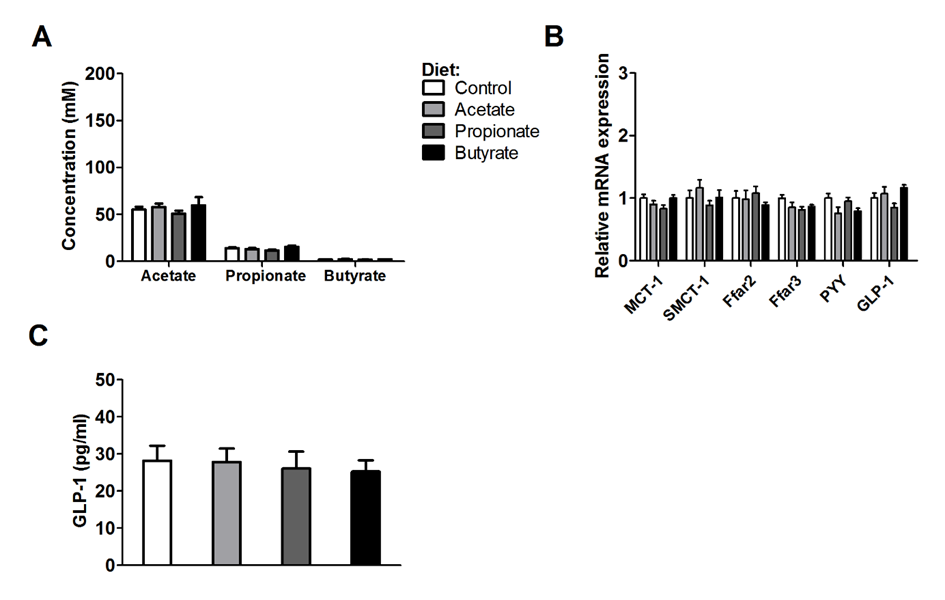

Supplement: S3 Fig — (DOCX) [file pone.0136364.s004.docx]
